# Integrating patient and whole-genome sequencing data to provide insights into the epidemiology of seasonal influenza A(H3N2) viruses

**DOI:** 10.1099/mgen.0.000137

**Published:** 2017-12-21

**Authors:** Emily J. Goldstein, William T. Harvey, Gavin S. Wilkie, Samantha J. Shepherd, Alasdair R. MacLean, Pablo R. Murcia, Rory N. Gunson

**Affiliations:** ^1^​ West of Scotland Specialist Virology Centre, NHS Greater Glasgow and Clyde, Glasgow, UK; ^2^​ Institute of Biodiversity, Animal Health and Comparative Medicine, University of Glasgow, Glasgow, UK; ^3^​ Medical Research Council-University of Glasgow Centre for Virus Research, Glasgow, UK

**Keywords:** influenza, surveillance, whole-genome sequencing, A(H3N2), reassortment, disease severity

## Abstract

Genetic surveillance of seasonal influenza is largely focused on sequencing of the haemagglutinin gene. Consequently, our understanding of the contribution of the remaining seven gene segments to the evolution and epidemiological dynamics of seasonal influenza is relatively limited. The increased availability of next-generation sequencing technologies allows rapid and economic whole-genome sequencing (WGS) of influenza virus. Here, 150 influenza A(H3N2) positive clinical specimens with linked epidemiological data, from the 2014/15 season in Scotland, were sequenced directly using both Sanger sequencing of the HA1 region and WGS using the Illumina MiSeq platform. Sequences generated by the two methods were highly correlated, and WGS provided on average >90 % whole genome coverage. As reported in other European countries during 2014/15, all strains belonged to genetic group 3C, with subgroup 3C.2a predominating. Multiple inter-subgroup reassortants were identified, including three 3C.3 viruses descended from a single reassortment event, which had persisted in the population. Cases of severe acute respiratory illness were significantly clustered on phylogenies of multiple gene segments indicating potential genetic factors warranting further investigation. Severe cases were also more likely to be associated with reassortant viruses and to occur later in the season. These results suggest that WGS provides an opportunity to develop our understanding of the relationship between the influenza genome and disease severity and the epidemiological consequences of within-subtype reassortment. Therefore, increased levels of WGS, linked to clinical and epidemiological data, could improve influenza surveillance.

## Data Summary

**Fig. 1.**
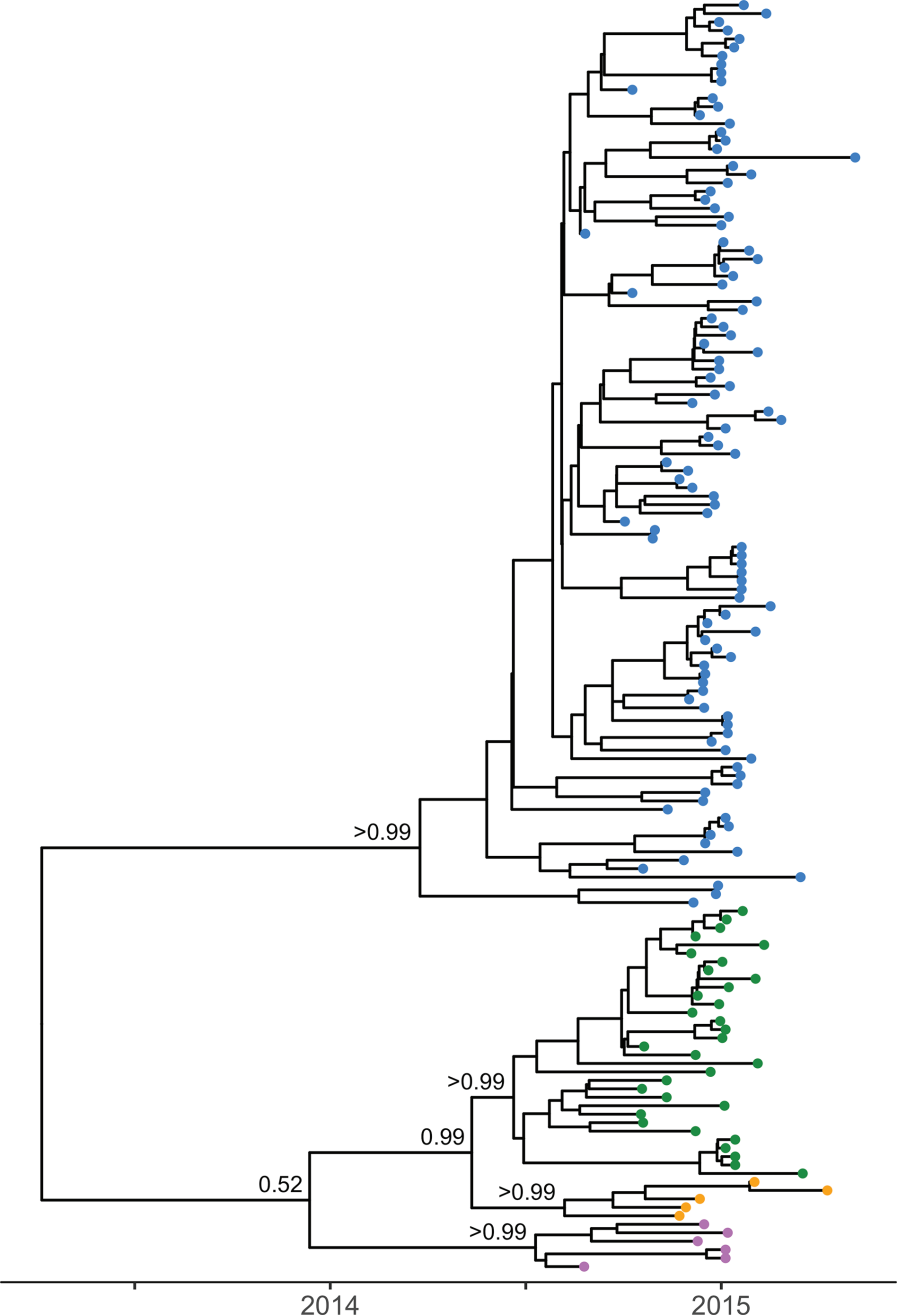
Phylogenetic tree of the haemagglutinin gene of influenza A(H3N2) viruses obtained from the 2014/15 influenza season. The MCC, time-resolved phylogeny for consensus sequences of the HA gene obtained using NGS. Genetic group is indicated by colour as follows: 3C.2a in blue, 3C.3 in orange, 3C.3a in pink and 3C.3b in green. Posterior probabilities associated with the nodes defining each of the four genetic groups were >0.99 (*n*=150).

1. Influenza genome sequences have been uploaded to the GISAID EpiFlu database (http://platform.gisaid.org). Accession numbers are listed in Table S1 (available in the online version of this article). These sequences are also available in fasta format on figshare: https://doi.org/10.6084/m9.figshare.5687284.

2. Tree files containing the maximum clade credibility phylogenetic trees plotted in Figs 1, 3 and 4 are provided in the following figshare fileset: https://doi.org/10.6084/m9.figshare.5433313.

3. Sample metadata consisting of GISAID ID, strain names, sample ID, date of sampling, week of sampling, Health Board, patient age, SARI status, sentinel surveillance status, reassortant status and the inferred genetic lineage of each gene segment as represented in Fig. 3 are available on figshare: https://doi.org/10.6084/m9.figshare.5427001.

**Impact Statement**

Each year, seasonal influenza infects up to 20 % of the human population, and evolving viral properties necessitate a global surveillance programme. The influenza genome consists of eight gene segments, which allows reassortment of viruses when cells are coinfected. Genetic surveillance is primarily focused towards the haemagglutinin gene, which encodes the principal target protein of the immune response. Consequently, relatively little is understood about the contributions of the other seven gene segments and reassortment to viral evolution, epidemiology and clinical outcomes. We demonstrate that whole-genome sequencing of influenza virus can be performed economically directly from clinical specimens and explore potential benefits over traditional sequencing regimes focused on haemagglutinin. Multiple reassortment events between genetic groups were identified, and we also observed a significant association between the most severe cases of respiratory illness and infection by these reassortant viruses. Furthermore, this work provides a useful framework for how whole-genome sequence data could be studied in combination with epidemiological data to aid surveillance of seasonal influenza.

## Introduction

Influenza viruses are a major cause of human morbidity and mortality worldwide, causing an estimated 250 000–500 000 deaths each year [1]. Influenza A virus (IAV) is an RNA virus, consisting of eight gene segments: RNA polymerase subunits polymerase basic 2 (PB2), polymerase basic 1 (PB1) and polymerase acidic (PA), haemagglutinin (HA), nucleoprotein (NP), neuraminidase (NA), matrix (M) and non-structural protein (NS). Classification of IAVs into subtypes is based on the combination of HA and NA they possess. IAVs evolve rapidly by both mutation and reassortment. Mutations conferring incremental selective advantages result in the characteristic rapid antigenic drift of IAVs, while the segmented nature of the genome allows genomic reassortment when a cell is coinfected by two or more strains. Inter-subtype reassortment occasionally gives rise to viruses with pandemic potential [2], the most recent of which emerged in 2009 [3]. Intra-subtype reassortment is likely to occur much more frequently and play an important role in increasing viral genetic diversity and adaptive potential [4].

Surveillance is required to ensure that vaccine components reflect the antigenic characteristics of circulating IAV strains [2]. The HA is the primary antigenic determinant and consequently the chief focus of genetic surveillance, with influenza viruses routinely characterized into genetic groups based on amino acid residues of the HA protein, as defined by the European Centre for Disease Prevention and Control (ECDC) [5]. Consequently, there is relatively little sequence data available for the remaining seven segments.

The West of Scotland Specialist Virology Centre (WoSSVC) is the Scottish influenza reference laboratory and is responsible for characterizing several hundred IAV-positive clinical specimens each year. Current characterization of IAVs by the WoSSVC is based on Sanger sequencing of the HA1 region of the HA gene. Next-generation sequencing (NGS) technology allows whole-genome sequencing (WGS) of IAVs in a single reaction, permitting rapid and economical sequencing with the potential for high throughput. In addition to viral characterization, WGS enables the detection of reassortment events and antiviral resistance mutations anywhere in the genome.

The 2014/15 influenza season in Scotland was dominated by influenza A(H3N2) [6], consistent with observations throughout the rest of the northern hemisphere that season [7]. All circulating influenza A(H3N2) viruses belonged to genetic group 3C; however, multiple lineages co-circulated throughout the season, with viruses of genetic subgroup 3C.2a predominating [7]. The majority of A(H3N2) viruses characterized were antigenically dissimilar to the A/Texas/50/2012-like vaccine virus (genetic group 3C.1), which may have contributed to the unusually high excess mortality observed during the 2014/15 influenza season [8].

The aim of this study was to assess the benefits of WGS over current Sanger sequencing methods for IAV surveillance and whether WGS can provide a greater understanding of the evolutionary and epidemiological dynamics of seasonal influenza. To this end, 150 influenza A(H3N2) positive clinical specimens from the 2014/15 influenza season in Scotland were sequenced using both methods. Genetic data and linked patient data were analysed to investigate rates of reassortment and potential associations between disease severity and phylogenies of each segment, reassortment status, and other patient details including location and age.

## Methods

### Samples

All 150 samples were influenza A(H3N2) positive clinical specimens submitted to the WoSSVC for routine influenza characterization. Inclusion criteria for the study were all samples collected between 1 August 2014 and 31 May 2015 which had previously been genetically characterized using Sanger sequencing, providing enough material was available. Samples were received from 11 Health Boards throughout Scotland and included throat swabs (*n*=85), combined nose and throat swabs (*n*=15), gargle (*n*=14), nasopharyngeal aspirate (*n*=11), sputum (*n*=6), nasal swabs (*n*=5), tracheal aspirate (*n*=2), bronchoalveolar lavage (*n*=1) and non-classified respiratory specimens (*n*=11). The samples selected for Sanger sequencing at WoSSVC included a selection of sentinel surveillance samples collected from general practice surgeries (*n*=16), samples from patients with severe acute respiratory illness (SARI), as defined by Health Protection Scotland (*n*=22), and 112 other clinical cases.

### RNA isolation

Nucleic acid was extracted directly from clinical samples using automated extraction methods. Swabs, gargles and nasopharyngeal aspirates were extracted using the BioRobot MDx (Qiagen) and sputum, tracheal aspirates and bronchoalveolar lavage were extracted using the NucliSENS easyMAG (bioMérieux). The same aliquot of extracted RNA was used for both Sanger sequencing and NGS.

### Sequencing of influenza viruses

Sanger sequencing was performed as previously described [9]. To prepare samples for NGS, RNA was reverse transcribed and the entire genome of influenza was amplified in a single RT-PCR reaction using the Uni/Inf primer set, as described by Zhou *et al*. [10]. Amplification was performed in 50 µl reactions containing 10 µl sterile water, 25 µl 2× RT-PCR buffer, 0.8 µl Uni12/Inf1 (10 µM), 1.2 µl Uni12/Inf3 (10 µM), 2 µl Uni13/Inf1 (10 µM), 1 µl SuperScript III Platinum Taq High Fidelity DNA Polymerase (Invitrogen) and 10 µl RNA. Thermocycling conditions were as follows: 42 °C for 60 min, 94 °C for 2 min; 5 cycles (94 °C for 30 s, 44 °C for 30 s and 68 °C for 3 min) followed by 31 cycles (94 °C for 30 s, 57 °C for 30 s and 68 °C for 3 min), with a final extension step at 68 °C for 5 min. DNA was diluted to a concentration of 175 ng in a volume of 50 µl and sheared acoustically using a Covaris S220 sonicator. NGS was performed on the Illumina MiSeq as previously described by Wilkie *et al*. [11], with the following modifications: DNA was purified using 0.9 volumes of AMPure XP beads; adapter-ligated DNA was amplified using six PCR cycles, and libraries were sequenced as 150 bp paired-end reads.

### Bioinformatics

Illumina adapter sequences were removed from the data, and paired-end reads were trimmed using a Phred score of 30 and to a minimum length of 50 bp using Trim Galore! (http://www.bioinformatics.babraham.ac.uk/projects/trim_galore/). Filtered reads were mapped to individual segments using the reference sequence A/Switzerland/9715293/2013. Two reference-mapping software packages were employed: Tanoti (http://www.bioinformatics.cvr.ac.uk/tanoti.php) and Bowtie2 (http://bowtie-bio.sourceforge.net/bowtie2/index.shtml); default settings were used for both packages. Files were converted to BAM format using SAMtools (http://samtools.sourceforge.net/), and consensus sequences were obtained using DiversiTools (http://josephhughes.github.io/DiversiTools/) using a majority rule and a minimum depth of one. Genome coverage and mean depth were greater using Tanoti; hence, all further analyses were performed using consensus sequences obtained using this tool. Consensus sequences have been uploaded to GISAID (http://platform.gisaid.org). Accession numbers are listed in Table S1.

### Phylogenetic analysis

Consensus nucleotide sequences were aligned using muscle [12]. Viruses were characterized into A(H3N2) genetic groups (subdivisions/subgroups) based on amino acid residues of the HA protein [5]. Time-resolved phylogenetic trees were reconstructed using BEAST v1.8.2 [13]. The general time reversible model with a proportion of invariant sites and a gamma distribution describing among-site rate variation with four categories estimated from the data (GTR +I+Γ_4_) was identified as the best model of nucleotide substitution through comparison of Bayes factors [14]. Bayes factor analysis also determined that a relaxed (uncorrelated) molecular clock model [15], with branch rates drawn from a lognormal distribution, and a minimally constrained Bayesian skyline demographic model [16] should be used. Chains were run until convergence as identified using Tracer v1.6.0 (available from http://tree.bio.ed.ac.uk/software/tracer/), and, after removing 10 % of trees as burn-in, a sample of posterior trees was analysed using TreeAnnotator v1.8.2 (available from http://beast.community/) to identify the maximum clade credibility (MCC) tree. The support for each node in the MCC tree is reflected by an associated posterior probability. Phylogenetic trees were visualised using the ggtree R package [17].

The positions of viruses of each genetic group were compared on phylogenies of each segment. Inconsistent positioning of single viruses, or groups of viruses, on these phylogenies was used to identify inter-subgroup reassortants. In addition to inconsistent positioning on MCC trees, posterior probabilities indicating high support for key nodes supporting inter-subgroup reassortment were required, to account for phylogenetic uncertainty. The rate of reassortment was estimated by dividing the number of reassortment events by the total number of viruses. Phylogenetic mapping of reassortants was also performed computationally using the Graph-incompatibility-based Reassortment Finder (GiRaF) software v1.02 [18]. Posterior samples of phylogenies generated for each segment using BEAST were thinned (downsampled) to 1000 trees and analysed to identify both inter- and intra-subgroup reassortment events. In principle, every reassortment event must split all eight segments into two subsets (retained and acquired segments); for some reassortment events the phylogenetic signal may be too weak for some segments to be assigned to one of the two reassorting sets of segments. Following the advice of the authors of the GiRaF software, a threshold of three pairwise comparisons resulting in at least four segments being placed confidently into either group was required. A confidence level of >0.95 was required for each pairwise comparison between segments that contributed to the detection of a reassortment event.

Bayesian Tip-association Significance (BaTS) analysis [19] was used to detect significant phylogeny-trait correlations, testing the null hypothesis that there is no correlation between phylogeny and trait. Each gene segment was tested for a phylogenetic association with severity of infection (classified into SARI and non-SARI), patient age (categorized into <1 year, 1–5 years, 6–15 years, 16–64 years and ≥65 years), location by Health Board and genetic subgroup. The association index (AI) was calculated for each tree in a posterior sample generated by BEAST (after excluding the first 10 % of tree states as burn-in) and compared with a null distribution generated by random reassignment of traits to tips of the phylogeny performed 5000 times. The AI ratio was calculated by dividing the observed AI by the null AI.

### Logistic regression

Predictors of severe infection (defined by classification of a SARI case) were investigated by logistic regression using R v3.3.2 [20]. Sentinel surveillance samples were excluded from this analysis; the remaining 134 samples included 22 SARI cases. Patient age measured in years, location by Health Board, the week of sampling, genetic subgroup and whether the virus was classified as a reassortant were tested as explanatory variables. To identify the best combination of explanatory variables, models were compared using Akaike information criterion (AIC) and likelihood ratio tests (LRT).

## Results

### Next-generation sequencing of influenza A(H3N2) directly from clinical specimens

The mean coverage across the entire influenza A(H3N2) genome was 91 %. Of the 150 viruses sequenced, complete genome coverage was achieved for 71 viruses and genome coverage of >90 % was generated for 100 viruses. Complete segment coverage was achieved for the two smallest segments, NS and M, in all 150 viruses. Segment coverage generally declined as the size of the segment increased; however, average segment coverage of ≥80 % was achieved for all segments (Table 1).

**Table 1.** Nucleotide coverage and depth of gene segments (*n*=150) of influenza A(H3N2) using NGS

Segment	Size (nucleotides)	Mean coverage	Mean depth	Number of samples with 100 % segment coverage
PB2	2280	80 %	1722	82
PB1	2274	83 %	1047	74
PA	2151	89 %	2226	105
HA	1701	96 %	2108	135
NP	1497	94 %	1844	126
NA	1410	98 %	2303	142
M	982	100 %	16 308	150
NS	838	100 %	16 712	150
Whole genome	13 133	91 %	3861	71

Using NGS, the mean coverage of the HA gene was 1646 nucleotides, compared with an average length of 551 nucleotides when the HA1 region was sequenced using the Sanger method. When sequences generated from Sanger and NGS were compared, >93 % of the viral sequences had ≤2 amino acid differences in HA1, demonstrating a good correlation in the sequences obtained using the two methods (full details of the number of discrepancies between Sanger and NGS at both nucleotide and amino acid levels are given in Table S2).

### Influenza A(H3N2) characterization, epidemiology and resistance using NGS data

Full segment coverage of HA was achieved for 90 % of samples using NGS, and sufficient sequence data was available to characterize all 150 samples into genetic groups according to ECDC guidelines [5]. All viruses belonged to genetic group 3C, of which 107 viruses (71 %) fell into genetic subgroup 3C.2a; five (3 %) into subdivision 3C.3; six (4 %) into subgroup 3C.3a; and 32 (21 %) within subgroup 3C.3b. Nodes defining these four genetic groups on the HA phylogeny were associated with posterior probabilities exceeding 0.99, indicating strong support for the phylogenetic distinctiveness of each (Fig. 1). As expected given the relatively small number of genetic differences in sequences obtained using NGS and Sanger sequencing, HA trees reconstructed using each method were broadly similar in topology, although a higher number of internal nodes were well supported (posterior probability >0.9) in the NGS HA tree (38 nodes) compared with the Sanger tree (24 nodes), indicating greater resolution when using NGS data.

**Fig. 2.**
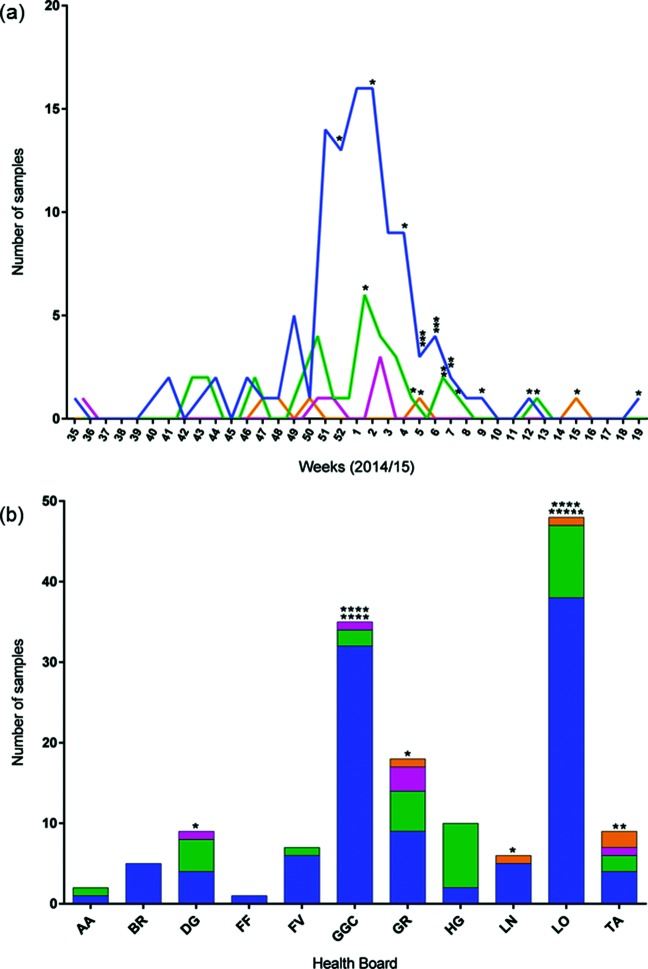
Circulation of influenza A(H3N2) viruses throughout the 2014/15 influenza season. (a) The number and genetic group of viruses received each week throughout the study period (*n*=150). (b) Health Board from which the samples were collected. Genetic groups are indicated by colour (3C.2a in blue, 3C.3 in orange, 3C.3a in pink and 3C.3b in green). AA, Ayrshire and Arran; BR, Borders; DG, Dumfries and Galloway; FF, Fife; FV, Forth Valley; GGC, Greater Glasgow and Clyde; GR, Grampian; HG, Highlands; LN, Lanarkshire; LO, Lothian; TA, Tayside. Each asterisk in (a) and (b) represents a case of SARI.

Viruses of each genetic group did not appear to cluster geographically or in time during the 10-month study period (Fig. 2), indicating co-circulation of distinct A(H3N2) lineages throughout the 2014/15 influenza season. While several A(H3N2) lineages did co-circulate, viruses of subgroup 3C.2a predominated both across the season as a whole and in each week during the peak of the epidemic season (Fig. 2a).

WGS enabled the presence or absence of drug resistance mutations in both the NA and the matrix 2 (M2) proteins to be determined. The S31N mutation in the M2 protein, which confers amantadine resistance, was present in all 150 viruses, consistent with previous studies [21]. Substitutions in NA resulting in resistance to neuraminidase inhibitors, E119V, D151E, I222V, R224K, E276D, R292K and R371K [22], were not detected (*n*=147).

### Analysis of whole-genome sequence data and identification of reassortments

**Fig. 3.**
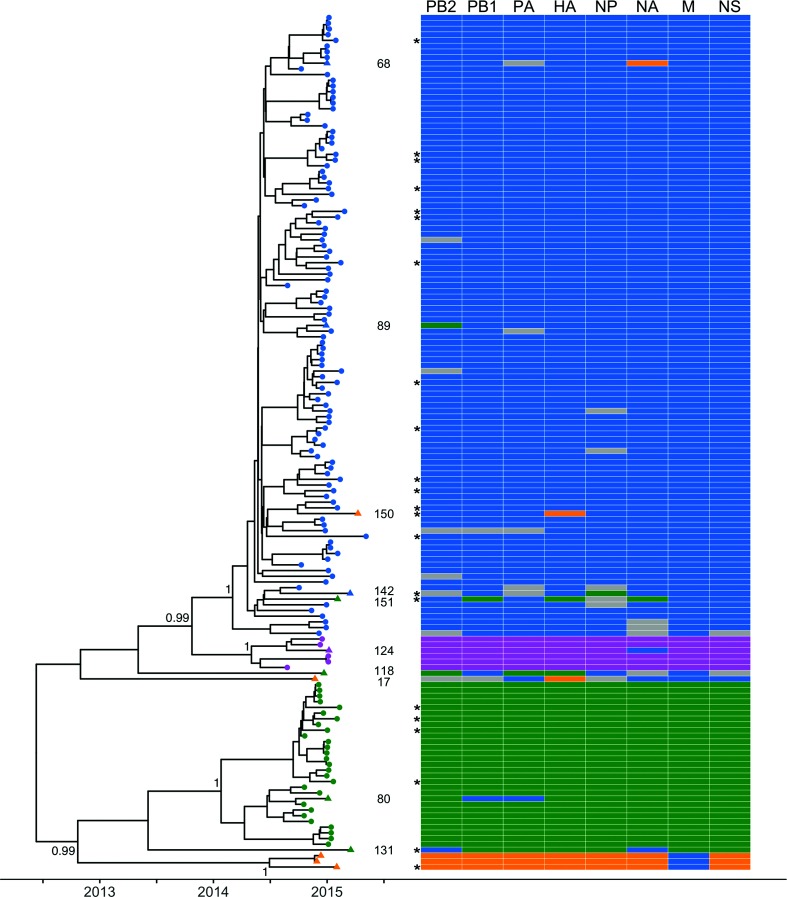
Phylogenetic tree of concatenated segments of influenza A(H3N2) viruses from the 2014/15 influenza season and schematic showing individual gene segment lineages. The MCC, time-resolved phylogenetic tree was reconstructed for the whole genome of influenza A(H3N2) by concatenating all eight segments (*n*=150). Tips are coloured by genetic group (as characterized by HA sequence) as follows: 3C.2a in blue, 3C.3 in orange, 3C.3a in pink and 3C.3b in green; triangles mark those identified as inter-subgroup reassortants (*n*=13). Sample numbers for these reassortants are also indicated; the three remaining unnumbered reassorted viruses make up the 3C.3 clade. Posterior probabilities associated with the nodes defining each of the four genetic groups were 1.00. Each asterisk represents a case of SARI. To the right, a schematic representation of viral clustering of each gene segment is shown. Where samples could not be confidently assigned to a genetic group phylogenetically for a particular segment, cells are coloured grey.

**Fig. 4.**
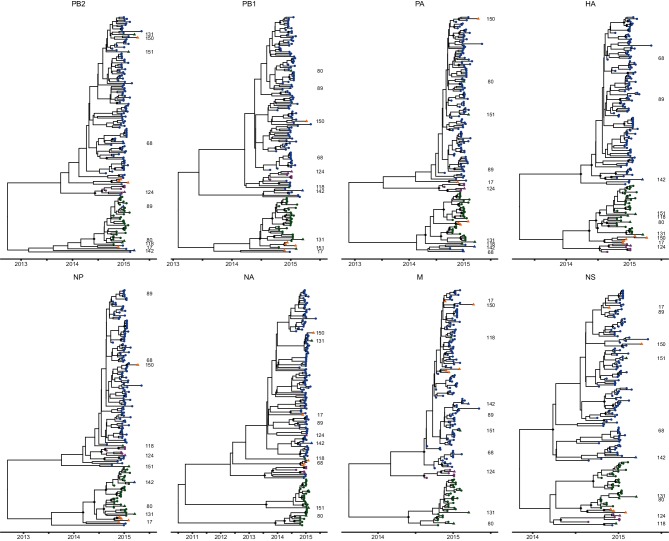
Phylogenetic trees of the eight individual gene segments of influenza A(H3N2) viruses from the 2014/15 influenza season. The MCC, time-resolved phylogenetic trees for consensus sequences of each segment obtained using NGS (*n*=150). Genetic groups are indicated on the tree by colour (3C.2a in blue, 3C.3 in orange, 3C.3a in pink and 3C.3b in green). The positions of novel inter-subgroup reassortant viruses in each phylogeny are indicated by triangles and are identifiable by sample number; the three remaining unnumbered reassorted viruses belong to the 3C.3 clade. Highly supported internal nodes of each phylogeny (posterior probability >0.9) are indicated by filled diamonds.

The sequences of all eight segments were concatenated to produce a single sequence for each virus and a whole-genome phylogenetic tree was reconstructed. The concatenated genomes revealed a topology which was generally consistent with that of the phylogenetic tree generated using the HA gene only, with posterior probabilities of 1.00 on the nodes defining each of the four clades corresponding to genetic subgroups 3C.2a, 3C.3, 3C.3a and 3C.3b (Fig. 3). The relatedness of genetic subgroup 3C.3a to the other clades is resolved in the full genome phylogeny (posterior probability=0.99) in contrast to the HA phylogeny (posterior probability=0.52, Fig. 1). Furthermore, a higher number of internal nodes possess posterior probabilities exceeding 0.9 (83 nodes), indicating greater phylogenetic resolution as a result of WGS. The full genome phylogeny also suggested the presence of some reassortant viruses, and to determine the evolutionary relationships between gene segments, eight individual phylogenetic trees were generated (Fig. 4). The position of viruses of each genetic group were compared on these phylogenies to identify inconsistencies arising from inter-subgroup reassortment.

Generally, viruses belonging to the same genetic group, as characterized by HA sequence, also clustered together on phylogenies generated from each of the remaining seven gene segments. This suggests an absence of reassortment in the recent evolutionary history of the majority of viruses. However, 13 viruses were identified to be inter-subgroup reassortants (marked with triangles in Figs 3 and 4). A schematic representation of the position in the phylogenies of each of the eight gene segments is shown alongside the full genome tree. Ten of the 13 inter-subgroup reassortants were identified as descending from reassortment events leading to individual viruses in this study. The remaining three inter-subgroup reassortants, characterized as genetic subdivision 3C.3, were inferred to descend from a further inter-subgroup reassortment event. With these 11 reassortment events, the rate of inter-subgroup reassortment is estimated to be 7.3 %.

Eight of these reassortment events were also detected using GiRaF, an automated reassortment detection tool (the rate of inter-subgroup reassortment is reduced to 5.3 % if only these events are considered). Of the remaining three reassortment events (samples 17, 118 and 150), samples 17 and 118 were identified by GiRaF but below the detection threshold chosen. Sample 118 was identified in one pairwise comparison as a single reassortant virus by GiRaF and twice as part of a reassortant clade, while sample 17 was identified once as a single reassortant and once as part of a reassortant clade. Uncertainty in the position of a virus on phylogenies of some segments may hinder detection by GiRaF by eroding confidence in inconsistencies; however, it may still be possible to identify inter-subgroup reassortants as the boundaries between genetic subgroups are identifiable. Viruses placed confidently within clades corresponding to a particular genetic subgroup on phylogenies of some segments and outside such clades on phylogenies of other segments can be considered inter-subgroup reassortments. For example, sample 150 is placed confidently in the 3C.3 subdivision on the HA tree (Fig. 1, posterior probability >0.99); however, in each of the other segment phylogenies it falls among 3C.2a viruses, and when the whole genome is considered it is placed among 3C.2a viruses with very high confidence (Fig. 3, posterior probability=1). Together, these posterior probabilities indicate support for inter-subgroup reassortment status. The GiRaF analysis additionally identified two intra-subgroup reassortment events in branches leading to two 3C.2a viruses and 18 3C.3b viruses, respectively. If all ten reassortment events identified using GiRaF (eight inter-subgroup and two intra-subgroup) are considered, the overall rate of reassortment is estimated to be 6.7 %.

The group of three reassortant viruses belonging to genetic subdivision 3C.3 formed a distinct clade in each of the individual segment phylogenies; however, the position of this clade in the overall topology varied. In seven of the eight segment trees, these viruses were placed either outside the clades of other subgroups or within the 3C.3b clade; however, in the M segment tree, the 3C.3 viruses cluster within the 3C.2a viruses (posterior probability >0.99) (Fig. 4). The M segments of these 3C.3 viruses were on average 10 and 14 nucleotides divergent from the M sequences of viruses belonging to genetic subgroups 3C.3a and 3C.3b, respectively, and differed by only four nucleotides on average from the M segment of 3C.2a viruses. The three clinical samples harbouring these viruses were collected between November 2014 and April 2015, demonstrating that this reassortant genotype has persisted in the population. To compare these 3C.3 viruses detected in Scotland with those observed in the rest of the United Kingdom, all UK whole-genome sequences from the 2014/15 influenza season available on GISAID (www.gisaid.org) were characterized genetically (*n*=171). Six of these viruses belonged to genetic subdivision 3C.3. Consistent with the pattern observed in the Scottish sequences, the M segment of these six viruses clustered amongst viruses characterized as genetic subgroup 3C.2a (data not shown).

### Analysis of virus phylogeny and trait correlations

To investigate predictors of a severe outcome in IAV infection, a logistic regression analysis was performed to analyse association between SARI cases and patient age, location, week of sampling, genetic subgroup and whether the virus was classified as reassortant. Week of sampling was found to be significantly correlated with severity of infection (LRT, χ^2^=62.2, df=1, *P*<1×10^−10^), with SARI cases more likely to occur later in the season (as seen in Fig. 2a). Severe cases were also found to be more likely to occur when patients were infected by a reassortant virus [LRT, χ^2^=4.9, df=1, *P*<0.05 for inter-subgroup reassortants (*n*=13) and χ^2^=6.1, df=1, *P*<0.05 for reassortant viruses identified using GiRaF (*n*=30)]. The odds ratio for the association between inter-subgroup reassortants and SARI cases was calculated as 4.4 (95 % confidence interval: 1.3–15.5), or 3.6 (1.3–9.7) when reassortants identified using GiRaF were considered. Model selection indicated that including reassortants identified using GiRaF in addition to week resulted in an improved model (χ^2^=5.16, df=1, *P*<0.05); however, including inter-subgroup reassortment status in addition to week did not (LRT, χ^2^=0.7, df=1, *P*>0.4). Further investigation showed that week of sampling was also correlated with inter-subgroup reassortment status (LRT, χ^2^=7.3, df=1, *P*<0.01); therefore, these could be confounding variables. No other explanatory variables tested (patient age, location or genetic subgroup) were found to be significant (Table S3).

BaTS analysis was used to test for a phylogenetic association with severity of infection, patient age, location and genetic group. Severity of infection was found to be strongly associated with the NP phylogeny (AI ratio=0.76, *P*<0.01), and slightly weaker correlations with the M, HA and NA phylogenies were also identified (*P*<0.05). Patient age was also found to be clustered to a greater degree on the NP gene phylogeny than expected by chance (AI ratio=0.90, *P*<0.05). Location and genetic group were strongly associated with phylogeny for all eight gene segments (*P*<1×10^−10^). These correlations indicate strong geographic clustering not apparent at the resolution of genetic subgroup (Fig. 2b) and that, as expected, viruses of the same genetic group (characterized by HA) tend to also have more similar sequences in other segments. BaTS analysis results are shown in full in Table S4.

## Discussion

The increased availability, and decreased costs and turn-around times, both for sequencing and data analysis, of NGS is revolutionizing microbiology. While Sanger sequencing of the HA1 region remains the predominant method used for IAV characterization globally, this study demonstrates the applicability of NGS technology for influenza surveillance, allowing WGS directly from clinical specimens. The benefits of WGS over existing Sanger sequencing protocols for IAV surveillance include: (1) greater resolution for genetic characterization of IAV; (2) the level of drug resistance mutations in the NA and M segments can be evaluated; (3) reassortment events can be detected and analysed; and (4) mutations in any region of the genome not yet understood to be important (e.g. virulence factors) are available for retrospective analysis. Such retrospective analysis of mutations in any region of the genome other than HA1 is not available using current surveillance methods.

We demonstrated effective WGS direct from clinical specimens using only one nucleic acid extraction, one RT-PCR and one NGS reaction. Many previous studies have propagated patient isolates in cell culture prior to sequence analysis [23]; however, the results presented herein suggest that this additional step is unnecessary. In addition to the reduced time and costs involved, direct sequencing methods allow for analysis of non-culturable strains and avoid unwanted mutations that have been shown to occur during viral propagation [23–25].

Using NGS, complete coverage of the HA gene was achieved for 90 % of samples and was adequate to allow characterization of 100 % of samples into genetic groups. When sequence data from Sanger and NGS were compared, high amino acid sequence homology was observed, providing further confidence that NGS could replace Sanger sequencing for routine influenza surveillance. Since 2016, ECDC guidelines have included HA2 residues for genetic characterization of both influenza A(H3N2) and A(H1N1)pdm09 [5]. This means additional Sanger sequencing reactions are required, increasing time and costs, whereas data for these HA2 residues are routinely available with WGS, strengthening the case for NGS further.

The data presented reveal spatiotemporal co-circulation of distinct viral lineages, supporting previous data suggesting that co-circulation of different A(H3N2) subgroups is common during epidemics of seasonal influenza [26–29]. This co-circulation facilitated inter-subgroup reassortment, estimated to occur at a rate of 5.3–7.3 % among the viruses studied. This is consistent with previous data suggesting that multiple reassortment events occur during an influenza season [28–31]; reported rates of within-subtype reassortment in seasonal A(H3N2) have ranged from 3 to 70 % [29, 31]. The level of inter-subgroup reassortment has been suggested to be an underestimate of true reassortment, as there may be undetected reassortment of segments between highly homogenous viruses of individual subgroups [32]. This was demonstrated here by the detection of additional intra-subgroup reassortments using computational detection methods. The 3C.3 lineage also demonstrates that inter-subgroup reassortment events can persist in the population and spread geographically. A total of nine viruses characterized as genetic subdivision 3C.3 (excluding novel reassortments) were observed (three and six from the Scottish and UK datasets, respectively) in 2014/15. In all of these viruses, the M segment clustered with viruses from genetic subgroup 3C.2a.

Persistence of intra-subtype reassortants has been demonstrated previously [33]. The factors associated with such persistence at a population level require further investigation; however, intra-subtype reassortment has been shown to temporarily raise the amino acid substitution rates contributing to an increased adaptive potential [34]. Specific examples of adaptive intra-subtype reassortment include a reassortment event between two antigenically distinct A(H3N2) lineages in 2003 that caused a major change in antigenic phenotype reducing vaccine effectiveness [26], and reassortment within A(H3N2) which has also led to the global rise and spread of resistance to adamantane drugs [35]. Increased WGS over consecutive influenza seasons would allow for an increased understanding of the frequency and timing of such intra-subtype reassortment and the contribution to the evolutionary dynamics of seasonal influenza.

Logistic regression analysis indicated that infection with a reassortant virus may be a risk factor for a severe outcome. It is possible that novel combinations of amino acids introduced by reassortment disrupt inter-gene co-adaptations, resulting in deviations from normal replication rates and virulence levels. With more data from WGS attached to patient information, this association could be investigated further. Both inter-subgroup reassortants and SARI cases were found to be more likely to occur later in the influenza season. It is possible that these correlations could result from a bias away from sampling milder cases later in the season. However, the distinct tendency for severe cases to occur later suggests that increased surveillance later in the season may be required to better understand the risk factors associated with disease severity. While the majority of A(H3N2) viruses circulating during the 2014/15 season were antigenically dissimilar to the vaccine virus [7], logistic regression did not show any relationship between genetic subgroup and the likelihood of a severe outcome. Therefore, there was no indication that subgroups varied in severity as a result of differing antigenic novelty. In future studies integrating sequence and patient data, it would be desirable to include patient vaccination history in order to further investigate the relationship between virus sequence, antigenic novelty and outcome of infection.

There are currently limited data in the literature regarding risk factors associated with disease severity [36]. Broberg *et al*. [37] recently recommended influenza sequence data to be reported along with epidemiological data to allow for greater definition of factors which may increase the risk of severe influenza. BaTS analysis identified a significant association between phylogenies generated from the NP, M, HA and NA gene segments and severe disease, with a particularly strong signal for NP. While these results should be interpreted with caution, they demonstrate the potential power of WGS coupled with linked epidemiological data. With more data, these correlations could be explored further to identify particular mutations in these genes which may be related to virulence.

In summary, this study demonstrates the benefit of NGS technology to provide whole-genome sequence data for surveillance of seasonal influenza viruses. The results of both the logistic regression and BaTS analysis emphasize that WGS coupled to linked patient data could be an important tool for developing our understanding of the relationship between the influenza genome and disease severity. More generally, WGS provides the opportunity to further investigate the epidemiological consequences of within-subtype reassortment and both the intra- and inter-season evolutionary dynamics of seasonal IAV at the whole-genome level.

## Data bibliography

1. Goldstein *et al.* GISAID EpiFlu database. http://platform.gisaid.org. Accession numbers are listed in Table S1 (2017).
2. Goldstein *et al.* Figshare. https://doi.org/10.6084/m9.figshare.5687284 (2017).
3. Goldstein *et al.* Figshare. https://doi.org/10.6084/m9.figshare.5433313 (2017).
4. Goldstein *et al.* Figshare https://doi.org/10.6084/m9.figshare.5427001 (2017).

## Supplementary Data

345 Supplementary File 1

## References

[1] World Health Organization (2016). Influenza (Seasonal) Fact Sheet 2016. www.who.int/mediacentre/factsheets/fs211/en/.

[2] Hay AJ, Gregory V, Douglas AR, Lin YP (2001). The evolution of human influenza viruses. Philos Trans R Soc Lond B Biol Sci.

[3] Dawood FS, Jain S, Finelli L, Shaw MW, Lindstrom S (2009). Emergence of a novel swine-origin influenza A (H1N1) virus in humans. N Engl J Med.

[4] Nelson MI, Holmes EC (2007). The evolution of epidemic influenza. Nat Rev Genet.

[5] European Centre for Disease Prevention and Control (2016). Influenza virus characterisation. Summary Europe, December 2015.

[6] Public Health England (2015). Surveillance of Influenza and Other Respiratory Viruses in the United Kingdom: Winter 2014 to 2015.

[7] World Health Organization (2015). Review of the 2014-2015 influenza season in the northern hemisphere. Wkly Epidemiol Rec.

[8] European Monitoring of Excess Mortality for Public Health Action (2015). Excess mortality in Europe in the winter season 2014/15, in particular amongst the elderly. Euro Surveill.

[9] Bradley-Stewart A, Miller RS, MacLean A, Aitken C, Whittaker L (2014). Cluster of influenza A cases in vaccinated population of adults in Virology Laboratory in Glasgow in December 2012. Scott Med J.

[10] Zhou B, Wentworth DE (2012). Influenza A virus molecular virology techniques. Methods Mol Biol.

[11] Wilkie GS, Davison AJ, Kerr K, Stidworthy MF, Redrobe S (2014). First fatality associated with elephant endotheliotropic herpesvirus 5 in an asian elephant: pathological findings and complete viral genome sequence. Sci Rep.

[12] Edgar RC (2004). MUSCLE: multiple sequence alignment with high accuracy and high throughput. Nucleic Acids Res.

[13] Drummond AJ, Suchard MA, Xie D, Rambaut A (2012). Bayesian phylogenetics with BEAUti and the BEAST 1.7. Mol Biol Evol.

[14] Suchard MA, Weiss RE, Sinsheimer JS (2001). Bayesian selection of continuous-time Markov chain evolutionary models. Mol Biol Evol.

[15] Drummond AJ, Ho SY, Phillips MJ, Rambaut A (2006). Relaxed phylogenetics and dating with confidence. PLoS Biol.

[16] Drummond AJ, Rambaut A, Shapiro B, Pybus OG (2005). Bayesian coalescent inference of past population dynamics from molecular sequences. Mol Biol Evol.

[17] Yu G, Smith DK, Zhu H, Guan Y, Lam TT-Y (2017). ggtree : an r package for visualization and annotation of phylogenetic trees with their covariates and other associated data. Methods Ecol Evol.

[18] Nagarajan N, Kingsford C (2011). GiRaF: robust, computational identification of influenza reassortments via graph mining. Nucleic Acids Res.

[19] Parker J, Rambaut A, Pybus OG (2008). Correlating viral phenotypes with phylogeny: accounting for phylogenetic uncertainty. Infect Genet Evol.

[20] R Core Team (2016). R: A language and environment for statistical computing.

[21] Li X, Liao H, Liu Y, Liu L, Wang F (2017). Drug-Resistant and Genetic Evolutionary Analysis of Influenza Virus from Patients During the 2013 and 2014 Influenza Season in Beijing. Microb Drug Resist.

[22] Eshaghi A, Shalhoub S, Rosenfeld P, Li A, Higgins RR (2014). Multiple influenza A (H3N2) mutations conferring resistance to neuraminidase inhibitors in a bone marrow transplant recipient. Antimicrob Agents Chemother.

[23] Mcwhite CD, Meyer AG, Wilke CO (2016). Sequence amplification via cell passaging creates spurious signals of positive adaptation in influenza virus H3N2 hemagglutinin. Virus Evol.

[24] Chambers BS, Li Y, Hodinka RL, Hensley SE (2014). Recent H3N2 influenza virus clinical isolates rapidly acquire hemagglutinin or neuraminidase mutations when propagated for antigenic analyses. J Virol.

[25] Tamura D, Nguyen HT, Sleeman K, Levine M, Mishin VP (2013). Cell culture-selected substitutions in influenza A(H3N2) neuraminidase affect drug susceptibility assessment. Antimicrob Agents Chemother.

[26] Holmes EC, Ghedin E, Miller N, Taylor J, Bao Y (2005). Whole-genome analysis of human influenza A virus reveals multiple persistent lineages and reassortment among recent H3N2 viruses. PLoS Biol.

[27] Nelson MI, Simonsen L, Viboud C, Miller MA, Taylor J (2006). Stochastic processes are key determinants of short-term evolution in influenza a virus. PLoS Pathog.

[28] Nelson MI, Edelman L, Spiro DJ, Boyne AR, Bera J (2008). Molecular epidemiology of A/H3N2 and A/H1N1 influenza virus during a single epidemic season in the United States. PLoS Pathog.

[29] Schweiger B, Bruns L, Meixenberger K (2006). Reassortment between human A(H3N2) viruses is an important evolutionary mechanism. Vaccine.

[30] Lindstrom SE, Cox NJ, Klimov A (2004). Genetic analysis of human H2N2 and early H3N2 influenza viruses, 1957–1972: evidence for genetic divergence and multiple reassortment events. Virology.

[31] Maljkovic Berry I, Melendrez MC, Li T, Hawksworth AW, Brice GT (2016). Frequency of influenza H3N2 intra-subtype reassortment: attributes and implications of reassortant spread. BMC Biol.

[32] Steel J, Lowen AC (2014). Influenza A virus reassortment. Curr Top Microbiol Immunol.

[33] Westgeest KB, Russell CA, Lin X, Spronken MI, Bestebroer TM (2014). Genomewide analysis of reassortment and evolution of human influenza A(H3N2) viruses circulating between 1968 and 2011. J Virol.

[34] Neverov AD, Lezhnina KV, Kondrashov AS, Bazykin GA (2014). Intrasubtype reassortments cause adaptive amino acid replacements in H3N2 influenza genes. PLoS Genet.

[35] Simonsen L, Viboud C, Grenfell BT, Dushoff J, Jennings L (2007). The genesis and spread of reassortment human influenza A/H3N2 viruses conferring adamantane resistance. Mol Biol Evol.

[36] Mertz D, Kim TH, Johnstone J, Lam P-P, Science M (2013). Populations at risk for severe or complicated influenza illness: systematic review and meta-analysis. BMJ.

[37] Broberg E, Hungnes O, Schweiger B, Prosenc K, Daniels R (2016). Improving influenza virological surveillance in Europe: strain-based reporting of antigenic and genetic characterisation data, 11 European countries, influenza season 2013/14. Euro Surveill.

