# Supplementary material for: Integrating patient and whole-genome sequencing data to provide insights into the epidemiology of seasonal influenza A(H3N2) viruses
Source: Microb Genom. 2017 Dec 21;4(1):e000137. doi: 10.1099/mgen.0.000137 (PMC5857367; doi:10.1099/mgen.0.000137)
Supplement: Supplementary File 1 [file mgen-4-137-s001.pdf]

Supplementary Materials

Strain name	GISAID Isolate ID
A/Scotland/1/2015 (H3N2)	EPI_ISL_285510
A/Scotland/10/2014 (H3N2)	EPI_ISL_286004
A/Scotland/100/2014 (H3N2)	EPI_ISL_286962
A/Scotland/101/2014 (H3N2)	EPI_ISL_286963
A/Scotland/102/2014 (H3N2)	EPI_ISL_286964
A/Scotland/103/2014 (H3N2)	EPI_ISL_286965
A/Scotland/104/2014 (H3N2)	EPI_ISL_286966
A/Scotland/105/2014 (H3N2)	EPI_ISL_286967
A/Scotland/106/2014 (H3N2)	EPI_ISL_286968
A/Scotland/107/2014 (H3N2)	EPI_ISL_286969
A/Scotland/108/2015 (H3N2)	EPI_ISL_286970
A/Scotland/109/2014 (H3N2)	EPI_ISL_286971
A/Scotland/11/2014 (H3N2)	EPI_ISL_286005
A/Scotland/110/2014 (H3N2)	EPI_ISL_286972
A/Scotland/111/2015 (H3N2)	EPI_ISL_286983
A/Scotland/112/2014 (H3N2)	EPI_ISL_286984
A/Scotland/113/2014 (H3N2)	EPI_ISL_286985
A/Scotland/114/2014 (H3N2)	EPI_ISL_286986
A/Scotland/115/2015 (H3N2)	EPI_ISL_286987
A/Scotland/116/2015 (H3N2)	EPI_ISL_286988
A/Scotland/117/2014 (H3N2)	EPI_ISL_286989
A/Scotland/118/2014 (H3N2)	EPI_ISL_286990
A/Scotland/119/2015 (H3N2)	EPI_ISL_286991
A/Scotland/12/2014 (H3N2)	EPI_ISL_286006
A/Scotland/120/2014 (H3N2)	EPI_ISL_286992
A/Scotland/121/2015 (H3N2)	EPI_ISL_286993
A/Scotland/122/2015 (H3N2)	EPI_ISL_286994
A/Scotland/123/2015 (H3N2)	EPI_ISL_286995
A/Scotland/124/2015 (H3N2)	EPI_ISL_286996
A/Scotland/125/2015 (H3N2)	EPI_ISL_286997
A/Scotland/126/2015 (H3N2)	EPI_ISL_286998
A/Scotland/127/2014 (H3N2)	EPI_ISL_286999
A/Scotland/128/2015 (H3N2)	EPI_ISL_287000
A/Scotland/129/2015 (H3N2)	EPI_ISL_287001
A/Scotland/13/2014 (H3N2)	EPI_ISL_286007
A/Scotland/130/2015 (H3N2)	EPI_ISL_287002
A/Scotland/131/2015 (H3N2)	EPI_ISL_287003
A/Scotland/132/2015 (H3N2)	EPI_ISL_287004
A/Scotland/133/2015 (H3N2)	EPI_ISL_287005
A/Scotland/134/2015 (H3N2)	EPI_ISL_287006
A/Scotland/135/2015 (H3N2)	EPI_ISL_287007
A/Scotland/136/2015 (H3N2)	EPI_ISL_287008
A/Scotland/137/2015 (H3N2)	EPI_ISL_287009
A/Scotland/138/2015 (H3N2)	EPI_ISL_287010
A/Scotland/139/2015 (H3N2)	EPI_ISL_287011
A/Scotland/14/2014 (H3N2)	EPI_ISL_286008

A/Scotland/140/2015 (H3N2)	EPI_ISL_287012
A/Scotland/141/2015 (H3N2)	EPI_ISL_287013
A/Scotland/142/2015 (H3N2)	EPI_ISL_287014
A/Scotland/143/2014 (H3N2)	EPI_ISL_287015
A/Scotland/144/2015 (H3N2)	EPI_ISL_287016
A/Scotland/145/2015 (H3N2)	EPI_ISL_287017
A/Scotland/146/2015 (H3N2)	EPI_ISL_287018
A/Scotland/147/2015 (H3N2)	EPI_ISL_287019
A/Scotland/148/2015 (H3N2)	EPI_ISL_287020
A/Scotland/149/2015 (H3N2)	EPI_ISL_287129
A/Scotland/15/2014 (H3N2)	EPI_ISL_286020
A/Scotland/150/2015 (H3N2)	EPI_ISL_287159
A/Scotland/151/2015 (H3N2)	EPI_ISL_287192
A/Scotland/16/2014 (H3N2)	EPI_ISL_286021
A/Scotland/17/2014 (H3N2)	EPI_ISL_286022
A/Scotland/18/2014 (H3N2)	EPI_ISL_286023
A/Scotland/19/2014 (H3N2)	EPI_ISL_286024
A/Scotland/2/2015 (H3N2)	EPI_ISL_285997
A/Scotland/20/2014 (H3N2)	EPI_ISL_286025
A/Scotland/21/2014 (H3N2)	EPI_ISL_286026
A/Scotland/22/2014 (H3N2)	EPI_ISL_286027
A/Scotland/23/2014 (H3N2)	EPI_ISL_286028
A/Scotland/24/2014 (H3N2)	EPI_ISL_286029
A/Scotland/25/2014 (H3N2)	EPI_ISL_286030
A/Scotland/26/2014 (H3N2)	EPI_ISL_286158
A/Scotland/27/2014 (H3N2)	EPI_ISL_286159
A/Scotland/28/2014 (H3N2)	EPI_ISL_286160
A/Scotland/29/2014 (H3N2)	EPI_ISL_286161
A/Scotland/3/2015 (H3N2)	EPI_ISL_285998
A/Scotland/30/2014 (H3N2)	EPI_ISL_286162
A/Scotland/31/2014 (H3N2)	EPI_ISL_286163
A/Scotland/32/2014 (H3N2)	EPI_ISL_286202
A/Scotland/33/2014 (H3N2)	EPI_ISL_286234
A/Scotland/34/2014 (H3N2)	EPI_ISL_286235
A/Scotland/35/2014 (H3N2)	EPI_ISL_286236
A/Scotland/36/2014 (H3N2)	EPI_ISL_286286
A/Scotland/37/2014 (H3N2)	EPI_ISL_286293
A/Scotland/38/2014 (H3N2)	EPI_ISL_286304
A/Scotland/39/2014 (H3N2)	EPI_ISL_286305
A/Scotland/4/2014 (H3N2)	EPI_ISL_285999
A/Scotland/40/2015 (H3N2)	EPI_ISL_286306
A/Scotland/41/2014 (H3N2)	EPI_ISL_286307
A/Scotland/42/2014 (H3N2)	EPI_ISL_286308
A/Scotland/43/2014 (H3N2)	EPI_ISL_286309
A/Scotland/44/2014 (H3N2)	EPI_ISL_286310
A/Scotland/45/2015 (H3N2)	EPI_ISL_286311
A/Scotland/46/2015 (H3N2)	EPI_ISL_286775
A/Scotland/47/2015 (H3N2)	EPI_ISL_286776
A/Scotland/48/2014 (H3N2)	EPI_ISL_286777
A/Scotland/49/2015 (H3N2)	EPI_ISL_286778

A/Scotland/5/2014 (H3N2)	EPI_ISL_286000
A/Scotland/50/2015 (H3N2)	EPI_ISL_286779
A/Scotland/51/2015 (H3N2)	EPI_ISL_286780
A/Scotland/52/2014 (H3N2)	EPI_ISL_286781
A/Scotland/53/2015 (H3N2)	EPI_ISL_286782
A/Scotland/54/2015 (H3N2)	EPI_ISL_286783
A/Scotland/55/2014 (H3N2)	EPI_ISL_286784
A/Scotland/56/2015 (H3N2)	EPI_ISL_286785
A/Scotland/57/2015 (H3N2)	EPI_ISL_286786
A/Scotland/58/2015 (H3N2)	EPI_ISL_286874
A/Scotland/59/2015 (H3N2)	EPI_ISL_286875
A/Scotland/60/2015 (H3N2)	EPI_ISL_286876
A/Scotland/61/2015 (H3N2)	EPI_ISL_286877
A/Scotland/62/2015 (H3N2)	EPI_ISL_286921
A/Scotland/63/2015 (H3N2)	EPI_ISL_286922
A/Scotland/64/2014 (H3N2)	EPI_ISL_286923
A/Scotland/65/2014 (H3N2)	EPI_ISL_286924
A/Scotland/66/2015 (H3N2)	EPI_ISL_286925
A/Scotland/67/2015 (H3N2)	EPI_ISL_286926
A/Scotland/68/2015 (H3N2)	EPI_ISL_286927
A/Scotland/69/2014 (H3N2)	EPI_ISL_286928
A/Scotland/7/2014 (H3N2)	EPI_ISL_286001
A/Scotland/70/2015 (H3N2)	EPI_ISL_286929
A/Scotland/71/2014 (H3N2)	EPI_ISL_286930
A/Scotland/72/2014 (H3N2)	EPI_ISL_286934
A/Scotland/73/2015 (H3N2)	EPI_ISL_286935
A/Scotland/74/2015 (H3N2)	EPI_ISL_286936
A/Scotland/75/2014 (H3N2)	EPI_ISL_286937
A/Scotland/76/2015 (H3N2)	EPI_ISL_286938
A/Scotland/77/2014 (H3N2)	EPI_ISL_286939
A/Scotland/78/2014 (H3N2)	EPI_ISL_286940
A/Scotland/79/2015 (H3N2)	EPI_ISL_286941
A/Scotland/8/2015 (H3N2)	EPI_ISL_286002
A/Scotland/80/2015 (H3N2)	EPI_ISL_286942
A/Scotland/81/2015 (H3N2)	EPI_ISL_286943
A/Scotland/82/2015 (H3N2)	EPI_ISL_286944
A/Scotland/83/2015 (H3N2)	EPI_ISL_286945
A/Scotland/84/2015 (H3N2)	EPI_ISL_286946
A/Scotland/85/2015 (H3N2)	EPI_ISL_286947
A/Scotland/86/2014 (H3N2)	EPI_ISL_286948
A/Scotland/87/2015 (H3N2)	EPI_ISL_286949
A/Scotland/88/2015 (H3N2)	EPI_ISL_286950
A/Scotland/89/2014 (H3N2)	EPI_ISL_286951
A/Scotland/9/2014 (H3N2)	EPI_ISL_286003
A/Scotland/90/2014 (H3N2)	EPI_ISL_286952
A/Scotland/91/2015 (H3N2)	EPI_ISL_286953
A/Scotland/92/2015 (H3N2)	EPI_ISL_286954
A/Scotland/93/2015 (H3N2)	EPI_ISL_286955
A/Scotland/94/2015 (H3N2)	EPI_ISL_286956
A/Scotland/95/2014 (H3N2)	EPI_ISL_286957

A/Scotland/96/2015 (H3N2)	EPI_ISL_286958
A/Scotland/97/2014 (H3N2)	EPI_ISL_286959
A/Scotland/98/2014 (H3N2)	EPI_ISL_286960

Table S1. Sample accession numbers. Sequences are available from GISAID (http://platform.gisaid.org) associated with the following accession numbers.

Number of discrepancies	Nucleotide level	Amino acid level
0	79 (53.0%)	93 (62.4%)
1	37 (24.8%)	36 (24.2%)
2	15 (10.1%)	10 (6.7%)
3	5 (3.4%)	3 (2.0%)
4	2 (1.3%)	0
5	3 (2.0%)	0
6	2 (1.3%)	2 (1.3%)
7	1 (0.7%)	1 (0.7%)
8	1 (0.7%)	0
9	0	1 (0.7%)
10	0	1 (0.7%)
12	0	1 (0.7%)
14	2 (1.3%)	0
15	1 (0.7%)	0
16	0	1 (0.7%)

21	1 (0.7%)	0
Mean discrepancies	1.32	0.88

Table S2. Number of discrepancies observed between Sanger and NGS sequences of HA1.

Model	AIC	χ^2	Degrees of freedom	p-value
Null intercept model	121.7	-	-	-
Single term models compared with null:				
Severity ~ **Age**	123.0	0.66	1	0.42
Severity ~ **Health Board**	128.1	9.61	8	0.29
Severity ~ **Genetic subgroup**	123.7	3.93	3	0.27
Severity ~ **Inter-subgroup reassortant**	118.8	4.85	1	0.028
Severity ~ **GiRaF reassortant**	117.5	6.14	1	0.013
Severity ~ **Week**	61.5	62.21	1	$1x10^{-10}$
Multi-term models compared with model including week only				
Severity ~ Week + **Age**	63.4	0.10	1	0.76
Severity ~ Week + **Inter-subgroup reassortant**	62.8	0.69	1	0.41
Severity ~ Week + **GiRaF**	58.3	5.16	1	0.023

**reassortant***				

Table S3. Logistic regression model quality as assessed by AIC and likelihood ratio tests. AIC scores for each model are shown, with smaller values indicating better quality. χ^2 values, degrees of freedom and p-values from likelihood ratio tests (LRTs) used to evaluate nested models are shown. The p-values are relevant for the emboldened and underlined term(s) in each model. An asterisk marks the best model as determined by comparison of AIC and using LRTs. Models including genetic subgroup or Health Board in addition to week could not be evaluated as fitted probabilities of 0 or 1 occurred, violating model assumptions.

Trait	Segment	Association index (AI) ratio[1]	*p*-value[2]
Severity of infection	PB2	0.96	0.37
	PB1	0.94	0.30
	PA	0.92	0.22
	HA	0.80	0.03
	NP	0.76	<0.01
	NA	0.80	0.03
	M	0.82	0.01
	NS	1.00	0.50
	PB2	0.92	0.07
	PB1	0.92	0.08
	PA	0.93	0.08

Age	HA	0.93	0.11
	NP	0.90	0.02
	NA	0.93	0.12
	M	0.95	0.11
	NS	0.94	0.09
Health Board	PB2	0.62	$<1x10^{-10}$
	PB1	0.63	$<1x10^{-10}$
	PA	0.71	$<1x10^{-10}$
	HA	0.62	$<1x10^{-10}$
	NP	0.70	$<1x10^{-10}$
	NA	0.62	$<1x10^{-10}$
	M	0.78	$<1x10^{-10}$
	NS	0.69	$<1x10^{-10}$
Genetic subgroup	PB2	0.16	$<1x10^{-10}$
	PB1	0.10	$<1x10^{-10}$
	PA	0.13	$<1x10^{-10}$
	HA	0.13	$<1x10^{-10}$
	NP	0.14	$<1x10^{-10}$
	NA	0.13	$<1x10^{-10}$
	M	0.11	$<1x10^{-10}$

	NS	0.13	$<1x10^{-10}$

Table S4. **Summary of results of BaTS analysis**. †Observed AI/null AI where null AI is derived from 5000 tree tip randomisations. Lower values indicate stronger phylogeny-trait associations in observed data. ‡BaTS null hypothesis test.
